# Down‐regulation of wheat Rubisco activase isoforms expression by virus‐induced gene silencing

**DOI:** 10.1002/pld3.583

**Published:** 2024-04-15

**Authors:** Juan Alejandro Perdomo, Joanna C. Scales, Wing‐Sham Lee, Kostya Kanyuka, Elizabete Carmo‐Silva

**Affiliations:** ^1^ Lancaster Environment Centre Lancaster University Lancaster UK; ^2^ School of Pharmacy and Biomedical Sciences University of Central Lancashire Preston UK; ^3^ Plant Biology and Crop Sciences Rothamsted Research Harpenden UK; ^4^ Biointeractions and Crop Protection Rothamsted Research Harpenden UK; ^5^ NIAB Cambridge UK

**Keywords:** co‐regulation, gene expression, protein isoforms, Rubisco activase, virus‐induced gene silencing (VIGS), wheat

## Abstract

Rubisco activase (Rca) is an essential photosynthetic enzyme that removes inhibitors from the catalytic sites of the carboxylating enzyme Rubisco. In wheat, Rca is composed of one longer 46 kDa α‐isoform and two shorter 42 kDa β‐isoforms encoded by the genes *TaRca1* and *TaRca2*. *TaRca1* produces a single transcript from which a short 1β‐isoform is expressed, whereas two alternative transcripts are generated from *TaRca2* directing expression of either a long 2α‐isoform or a short 2β‐isoform. The 2β isoform is similar but not identical to 1β. Here, virus‐induced gene silencing (VIGS) was used to silence the different *TaRca* transcripts. Abundance of the transcripts and the respective protein isoforms was then evaluated in the VIGS‐treated and control plants. Remarkably, treatment with the construct specifically targeting *TaRca1* efficiently decreased expression not only of *TaRca1* but also of the two alternative *TaRca2* transcripts. Similarly, specific targeting of the *TaRca2* transcript encoding a long isoform *TaRca2α* resulted in silencing of both *TaRca2* alternative transcripts. The corresponding protein isoforms decreased in abundance. These findings indicate concomitant down‐regulation of *TaRca1* and *TaRca2* at both transcript and protein levels and may impact the feasibility of altering the relative abundance of Rca isoforms in wheat.

## 1 INTRODUCTION

Rubisco is responsible for the net CO_2_ assimilation through the carboxylation of ribulose‐1,5‐bisphosphate (RuBP). However, Rubisco is prone to inhibition by the unproductive binding of sugar‐phosphates that lock the catalytic sites in a closed conformation. One of its inhibitors is the substrate, RuBP, which can bind the uncarbamylated sites of Rubisco (Brooks & Portis, 1988; Jordan & Cholletz, 1983; Portis, 1995). Rubisco activase (Rca) is a catalytic chaperone of Rubisco and part of the AAA+ protein family, which uses the energy from ATP hydrolysis to remodel the conformation of Rubisco (Bhat et al., 2017; Mueller‐Cajar, 2017; Portis, 2003). This ATPase activity restores the catalytic competence of Rubisco by promoting the release of inhibitory sugar‐phosphates from the Rubisco catalytic sites.

In most vascular plant species, Rca is composed of two isoforms that are identical, except for a 30–39 amino acid extension at the C‐terminus that differentiates the α long isoform from the β short isoform (Nagarajan & Gill, 2018; Salvucci et al., 1987; Werneke et al., 1989). The Rca α and β isoforms are the product of either alternative splicing or separate genes depending on the species. In *Arabidopsis thaliana* and *Spinacia oleracea* L. (spinach) the alternative splicing of a single gene produces both Rca isoforms (Werneke et al., 1989). However, in *Hordeum vulgare* L. (barley), two genes are present; one *Rca* gene is alternatively spliced to produce two mRNAs that encode the α long isoform and β short isoform, while the second gene produces only the β short isoform (Rundle & Zielinskis, 1991). The abundance of the two Rca isoforms—α and β—also varies considerably among species, with the amount of the α isoform sometimes equal but generally much less than the β isoform (Degen et al., 2021; Harvey et al., 2022; Kim et al., 2021; Perdomo et al., 2021; Salvucci et al., 1987). There are also some species, like *Nicotiana tabacum* (tobacco), which are known to only produce the β short isoform (Ayala‐Ochoa et al., 2004; Wang et al., 1992).

In *Triticum aestivum* L. (wheat) the two genes *TaRca1* and *TaRca2*, located in tandem in chromosome 4, encode three Rca isoforms; one α long isoform and two β short isoforms. Expression of *TaRca1* produces a short isoform TaRca1β protein (42.7 kDa) only, whereas alternative splicing of *TaRca2* results in either a long isoform TaRca2α (46 kDa) or a short isoform TaRca2β (42.2 kDa) (Carmo‐Silva et al., 2015). It has been shown that TaRca1β is the least abundant of the three Rca isoforms representing only 1% of the Rca pool, while TaRca2β is the most abundant accounting for 84% of the Rca pool in wheat leaves (Degen et al., 2021).

Altering the expression of *Rca* can affect the expression and abundance of Rubisco, with some studies in rice showing that *Rca* overexpression results in reduced abundance of Rubisco and impairment of photosynthesis (Fukayama et al., 2018; Suganami et al., 2020). Likewise, different studies have shown that *Rca* expression fluctuates during the diurnal cycle with the abundance of *Rca* transcripts having circadian rhythms in some species (Martino‐Catt & Ort, 1992; Pilgrim & McClung, 1993; To et al., 1999; Watillon et al., 1993). In wheat, the expression of the different *Rca* transcripts fluctuates during the diurnal cycle and *TaRca1* is expressed at very low levels compared with *TaRca2* (Perdomo et al., 2021). The wheat Rca protein isoforms differ in their regulatory properties, displaying different sensitivities to ADP inhibition (Perdomo et al., 2019; Scafaro, De Vleesschauwer, et al., 2019). Moreover, the wheat Rca isoforms also differ in their response to temperature, with 1β showing greater tolerance to elevated temperatures (Scafaro, Bautsoens, et al., 2019; Degen et al., 2020).

The above findings suggest that altering the relative expression of Rca isoforms could be pursued to improve the regulation and thermal tolerance of photosynthesis (Amaral et al., 2023; Qu et al., 2023; Sparrow‐Muñoz et al., 2023; Wijewardene et al., 2021). Despite very similar peptide identities between the Rca isoforms in wheat, differences in the *TaRca* transcript sequences (Carmo‐Silva et al., 2015) were explored to use virus‐induced gene silencing (VIGS) to knock‐down the expression of the individual transcripts and assess the impact on the abundance of the other transcripts and the corresponding protein isoforms.

VIGS is a reverse genetic tool that takes advantage of the natural, conserved RNA interference (RNAi) antiviral defense response operating in plants for rapid silencing of endogenous genes to aid dissection of their function (Lu et al., 2003; Purkayastha & Dasgupta, 2009). VIGS is popular as it is simple, often involving agroinfiltration or biolistic inoculation of plants, and relatively inexpensive. Moreover, results are obtained rapidly, typically within 2–3 weeks after treatment, and the technology bypasses stable plant transformation steps and therefore is applicable to numerous plant species, including those recalcitrant to genetic transformation (Lu et al., 2003; Purkayastha & Dasgupta, 2009). VIGS is mediated by small interfering RNAs (siRNAs) in a sequence specific manner. By inserting a fragment of a plant gene into a cloned virus genome, transcripts of the gene expressed by the plant become targets for degradation, therefore causing the gene of interest to be significantly down‐regulated or knocked‐down at the transcript level (Lee et al., 2012; Unver & Budak, 2009). This approach allows phenotypes resulting from silencing the genes of interest to be observed.

**FIGURE 1:**
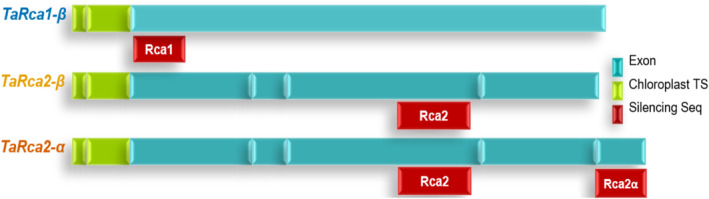
Schematic representation of wheat Rubisco activase (TaRca) genes highlighting the three regions targeted by the VIGS constructs. *Note:* Diagram of the three *TaRca* transcripts from wheat (*TaRca1β*, *TaRca2β*, and *TaRca2α*) and the three regions targeted using VIGS indicated in red below the corresponding *TaRca* transcript.

We generated three VIGS constructs to silence the two wheat *TaRca* genes and their alternative transcripts (Figure 1). The first construct was designed to silence *TaRca1* and reduce the production of TaRca1β. The second construct targeted *TaRca2* for silencing, aiming to silence both alternative transcripts and thus reduce abundance of the corresponding TaRca2α and TaRca2β isoforms. The third construct also targeted *TaRca2* but was designed to specifically silence the longest of the two alternative transcripts, hence decreasing production of only the TaRca2α isoform. The levels of the respective transcripts and the abundance of TaRca2α and the two TaRcaβ protein isoforms were evaluated in the VIGS‐treated plants and control plants using qRT‐PCR and immunoblotting, correspondingly. The aim of this study was to characterize the expression levels and protein abundance of the three Rca isoforms following treatments with the three different VIGS constructs, thereby gaining valuable new insights to inform future strategies to engineer wheat plants with altered abundance of Rca isoforms.

## 2 MATERIAL AND METHODS

### 2.1 VIGS of Rca in wheat

The *Barley stripe mosaic virus* (BSMV) vector was used to generate three VIGS constructs, BSMV::Rca1, BSMV::Rca2, and BSMV::Rca2α, to silence the *TaRca1β*, *TaRca2β/α*, and *TaRca2α* transcripts in wheat, respectively. Likewise, one negative control VIGS construct containing a 250–400 nt fragment of a non‐plant origin gene, in this case the *Aequorea victoria Green Fluorescent Protein gene* (BSMV::asGFP; GenBank accession E17099), was used in this study. The BSMV vectors described by Yuan et al. (2011), comprising three T‐DNA binary plasmids, pCaBS‐α, pCaBS‐β, and pCa‐ɣbLIC, were utilized for the VIGS constructs mentioned above.

siRNA‐Finder (si‐Fi) (Lück et al., 2019) was used to predict gene‐specific regions in the *TaRca1* and *TaRca2* transcript sequences that would produce the highest number of silencing‐efficient siRNAs and to check against the wheat genome (IWGSC RefSeq v1.0) that non‐target genes were unlikely to be silenced (Figures S1 and S2). Using this information, a 117 bp region in the *TaRca1* transcript and 189 and 108 bp regions in the *TaRca2* transcripts were selected for use in silencing constructs and primers were designed (Table 1) to amplify these regions by RT‐PCR from the total wheat RNA.

**TABLE 1:** Primers used for amplification of *TaRca* gene fragments for VIGS construct preparation.

*TaRca* silencing fragment	Primer sequences
BSMV::Rca1β	
Forward	AAC CAC CAC CAC CGT GCC AAA AAG GAA CTT GAC GAG
Reverse	AAG GAA GTT TAA GGA GTC CAC GAT ACC TTT CC
BSMV::Rca2	
Forward	AAC CAC CAC CAC CGT AAG GAG GAG AAC CCT CGT GTG
Reverse	AAG GAA GTT TAA GAC GAT CTT GAC GAC GGA CTC
BSMV::Rca2α	
Forward	AAC CAC CAC CAC CGT GCA CAG CAA GGT ACT TTG CCT GT
Reverse	AAG GAA GTT TAA TTA AAA GGT GTA AAG GCA GCT SCC G

*Note*: Constructs were designed for silencing of *TaRca1* (BSMV::Rca1β), both the *TaRca2‐α* and *TaRca2‐β* alternative transcripts (BSMV::Rca2), and the *TaRca2‐α* transcript only (BSMV::Rca2α).

Total RNA was extracted from leaves of young seedlings of wheat cv. Cadenza using the TRIzol reagent (Invitrogen, Life Technologies, UK) following the manufacturer's instructions. The total wheat RNA was converted to the first‐strand cDNA using oligo (dT)_20_ primers and reverse transcriptase SuperScript®III (Invitrogen, Life Technologies, UK). Regions of *TaRca1* and *TaRca2* for use in VIGS were amplified using the first‐strand wheat cDNA as a template and Phusion DNA polymerase (New England BioLabs Inc., UK). PCR conditions were 98°C for 30 s followed by 40 cycles of 98°C for 10 s, 70°C for 10 s, and 72°C for 10 s and a final extension step at 72°C for 5 min.

The above PCR products were cloned into the pCa‐γbLIC plasmid using ligation independent cloning (LIC), then, the BSMV vectors were transformed in *Agrobacterium tumefaciens* cells by electroporation as described by Panwar and Kanyuka (2022). Suspensions of the *A. tumefaciens* strains transformed individually with pCaBS‐α, pCaBS‐β, and pCa‐ɣbLIC were then mixed together in a 1:1:1 ratio and infiltrated into the abaxial side of 3–4 weeks old *Nicotiana benthamiana* plants with a 1 mL needleless syringe. Three to 4 days post‐infiltration, once virus symptoms were visible on *N. benthamiana* infected leaves, the infiltrated leaves were harvested, and ground in 10 mM potassium phosphate buffer (pH 6.8) containing 1% Celite 545, acid‐washed (Fisher Scientific UK Ltd.). This homogenate was used to mechanically treat the first leaf of 11‐day‐old wheat seedlings (cv. Cadenza). At 14 days post treatment, upper uninoculated leaf samples showing BSMV symptoms were harvested for both qRT‐PCR and western blotting. A 10 cm length of leaf tissue was harvested from the fourth leaf. Samples were collected 5–7 h into the light period to ensure maximal TaRca protein expression. The experiment was carried out twice and the results showed no significant differences among the replicated experiments (Table S1). Therefore, the data from the two independent experiments is presented together.

### 2.2 Gene expression determination

Expression of the *TaRca1* and *TaRca2α* and *TaRca2β* transcripts was determined by qRT‐PCR. Total RNA extraction from the experimental plants was carried out using the hot phenol method (Shinmachi et al., 2010; Verwoerd et al., 1989), and cDNA was synthesized using oligo (dT)_20_ and Superscript III as per the manufacturer's instructions (Life Technologies Ltd., UK).

qRT‐PCR conditions (Table S2) and transcript expression quantification were done as described in Perdomo et al. (2021). Three primer pairs were used for qRT‐PCR (Table 2) with each pair specific to the particular Rca isoform encoding transcripts across all three wheat sub‐genomes (A, B, and D). In the case of *Rca2β* and *Rca2α*, the design of isoform‐specific primers took advantage of the alternative splicing event at the end of *Rca2β* (Carmo‐Silva et al., 2015). The forward primer for *Rca2β* is in the 3′‐untranslated region (UTR) for the transcript; this is not present in *Rca2α* because that part of the sequence is in the last intron for the *Rca2α* transcript. The forward primer for *Rca2α* spans the end of Intron 5 and the start of Intron 6 and thus is specific for *Rca2α* transcripts. The reverse primer is common to both transcripts and located in the 3′‐UTR for *Rca2β* or the last exon for *Rca2α*. Wheat *cell division cycle protein 48* (*TaCDC48*) and *tonoplast intrinsic protein* (*TaTIP41*) were used as reference genes. Eight biological replicates were collected for each VIGS construct, and the experiment was repeated twice under exactly the same conditions.

**TABLE 2:** Sequences of qRT‐PCR primer pairs used for measuring *TaRca* gene expression in wheat plants treated with VIGS constructs.

Gene	Gene ID	Primer	Primer sequences
*TaRca1β*	TraesCS4A02G177600 TraesCS4B02G140200 TraesCS4D02G134900	Forward	GGG TCG GCG AGA TCG GCG T
		Reverse	CCA GCA TGT GGC CGT ACT CCA TG
*TaRca2β*	TraesCS4A02G177500 TraesCS4B02G140300 TraesCS4D02G135000	Forward	CCA TAC ACA CCC ACC ATC TCT TGC
		Reverse	TGT AAA GGC AGC TCC CGT CGT
*TaRca2α*		Forward	CCT TCT ACG GTA AAG GGG CAC AG
		Reverse	TGT AAA GGC AGC TCC CGT CGT
*TaTIP41*	TraesCS5A02G398100 TraesCS5B02G403200 TraesCS5D02G407600	Forward	TGC AGC AAA ATG GAA ATT CA
		Reverse	TGC GTA GCA TCT TGG TTC AG
*TaCDC48*	TraesCS5A02G301500 TraesCS5B02G299200 TraesCS5D02G306600	Forward	GTC CTC CTG GCT GTG GTA AAA
		Reverse	AGC AGC TCA GGT CCC TTG ATA

*Note*: Wheat *tonoplast intrinsic protein* (*TaTIP41*) and *cell division cycle protein 48* (*TaCDC48*) were used as reference genes.

### 2.3 Quantification of the TaRca isoforms and Rubisco protein abundance

The abundance of the Rca isoforms and Rubisco protein in the VIGS‐treated and control wheat plants was determined according to Perdomo et al. (2018). The total soluble protein (TSP) amount in each sample was measured using Bradford reagent (Bradford, 1976), and denatured samples were then diluted based upon TSP to a concentration of 1 mg mL^−1^ with SDS loading buffer. Sample volumes corresponding to 3 μg TSP were run on hand‐cast 15% SDS‐PAGE gels and either visualized by staining with Coomassie Blue for Rubisco (Figure S3A) or subjected to immunoblotting for Rca (Figure S3B). For the latter, a primary antibody against cotton Rca produced in rabbit (Salvucci, 2008) and a fluorescent secondary antibody were used for visualization of Rca using an Odyssey Fc imaging (LI‐COR, Lincoln, USA). For Rca and Rubisco quantification on each gel, standard calibration curves were generated with a dilution series (.1, .25, .5, 1.0, and 1.2×) from the pool of BSMV::asGFP (a negative control)‐treated samples.

### 2.4 Data analysis

Kruskal–Wallis was used to test the statistical significance in gene expression and protein abundance among the different constructs. A post hoc test using the Fisher's least significant difference criterium was used for multiple pairwise comparisons. A two‐way ANOVA was used to test for significant differences between the data obtained from the two independent experiments. Data were analyzed using R 3.6.2 (R Core Team, 2020), RSTUDIO 1.2.5033 (RStudio Team, 2020) and the agricolae R package 1.4.0 for analysis of variance (Mendiburu & Yaseen, 2020). Linear model (Lm) was used to predict the concentration of protein based on the calibration curve. BioEdit (Hall, 1999) was used to prepare the sequence alignments (Figure S1).

## 3 RESULTS

Three different silencing constructs were developed to specifically reduce the relative abundance of the Rca isoforms using VIGS. The first construct (BSMV::Rca1) was designed to specifically silence *TaRca1* and thus decrease the abundance of TaRca1β. The second construct (BSMV::Rca2) was predicted to silence both *TaRca2α* and *TaRca2β*, and the third construct (BSMV::Rca2α) was designed to target the *TaRca2* alternative transcript coding for the TaRca2α isoform (Figures 1 and S1). Control plants were treated with the construct BSMV::asGFP, which does not have any silencing targets in the wheat genome.

**FIGURE 2:**
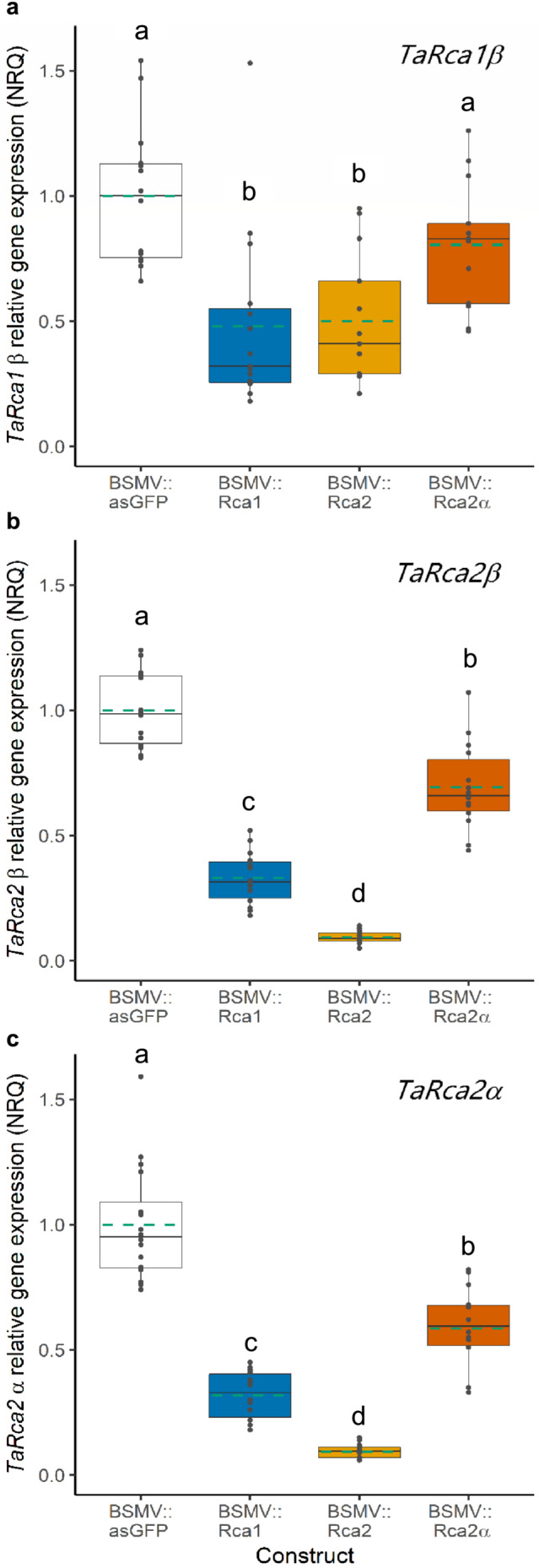
Relative expression of the three *TaRca* transcripts in plants treated with the different VIGS constructs. *Note*: Normalized relative quantity (NRQ) of the (a) *TaRca1β*, (b) *TaRca2β*, and (c) *TaRca2α* transcripts in wheat leaf tissue sampled from the VIGS‐treated and control plants. Expression of the three Rca‐encoding transcripts in the negative control, BSMV::asGFP‐treated plants, was set to 1. Gene expression was estimated as NRQ using *TaTIP41* and *TaCDC48* as reference genes. Boxes represent the median and the first and third quartiles, and whiskers represent the range; symbols represent individual samples and dashed green lines represent the mean (*n* = 14–16 biological replicates from two experiments). Kruskal–Wallis test showed significant effects of BSMV::Rca constructs on the expression of the three *TaRca* transcripts (*P* < .001). Different letters denote significant differences between the control and the three BSMV::Rca constructs for each isoform (post hoc test uses the criterium Fisher's least significant difference, *P* < .05).

Primers specific for each of the three *TaRca* transcripts were used to determine their expression levels by qRT‐PCR in plants inoculated with each of the silencing constructs (Table 2) and in control BSMV::asGFP inoculated plants. As expected, reduced expression of each of the three target transcripts was observed in plants treated with the corresponding VIGS constructs, in comparison with the BSMV::asGFP‐treated control plants. Although BSMV::Rca1 was designed to specifically silence *TaRca1* (Figures 1, S1, and S2), plants inoculated with BSMV::Rca1 accumulated low levels not only of *TaRca1* but also of the two *TaRca2* transcripts (Figure 2a–c). As anticipated, the BSMV::Rca2‐treated plants accumulated very low levels of *TaRca2α* and *TaRca2β* transcripts; however, unexpectedly, these same plants also accumulated lower levels of *TaRca1β* compared with the negative control plants (BSMV:asGFP). Also, while BSMV::Rca2α was designed to silence *TaRca2α* specifically, we observed lower levels of both *TaRca2α* (Figure 2c) and *TaRca2β* (Figure 2b) transcripts in these plants, while expression of *TaRca1β* was slightly lower albeit not significantly different compared with the control plants (Figure 2a).

**FIGURE 3:**
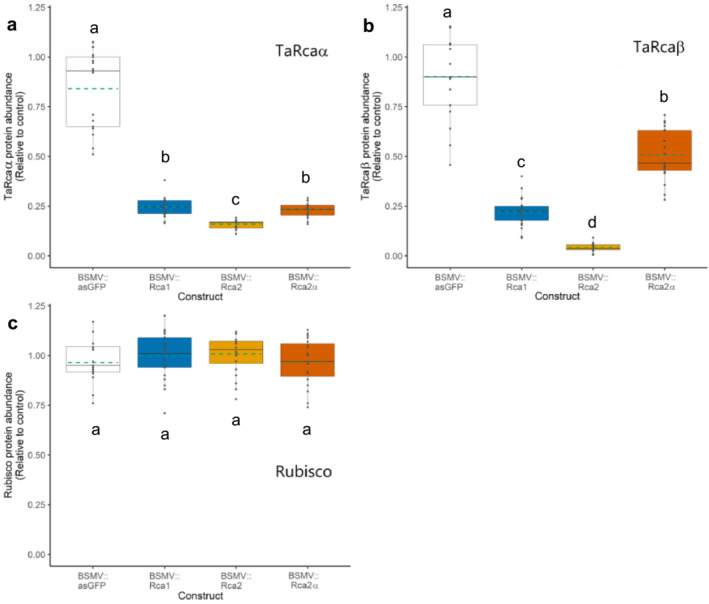
Relative abundance of TaRcaα, TaRcaβ, and Rubisco proteins in plants treated with the different VIGS constructs. *Note:* Relative protein amounts of (a) TaRcaα, (b) TaRcaβ, and (c) Rubisco large subunit in wheat leaves of plants inoculated with BSMV::Rca silencing constructs compared with the control (BSMV::asGFP). Rca amount was estimated by reference to a calibration curve prepared with increasing amounts of the control BSMV::asGFP samples. Boxes represent the median and the first and third quartiles, and whiskers represent the range; symbols represent individual samples and dashed green lines represent the mean (*n* = 14–16 biological replicates from two experiments). Kruskal–Wallis test showed significant effects of BSMV::Rca constructs in the abundance of the *TaRca* isoforms (*P* < .001), but no significant effects were found on the Rubisco amount. Different letters denote significant differences between the control and the three BSMV::Rca constructs (post hoc test uses the criterium Fisher's least significant difference, *P* < .05).

To determine whether the lower expression of the *TaRca* genes induced by VIGS translated into lower Rca protein abundance, immunoblotting analysis was carried out to quantify the relative abundance of the TaRca isoforms. Isoforms TaRca1β and TaRca2β had to be quantified together due to their very similar molecular masses of 42.7 and 42.2 kDa, respectively (Carmo‐Silva et al., 2015). The immunoblotting analysis showed decreased abundance of both the longer TaRcaα and the shorter TaRcaβ isoforms in all plants, regardless of the VIGS construct used. The extent of the decrease was more pronounced in plants inoculated with BSMV::Rca2, designed to silence the *TaRca2α* and *TaRca2β* transcripts (Figure 3a,b), where protein abundance levels were less than 5% for TaRcaβ and less than 20% for TaRcaα compared with those observed in control plants. The abundance of TaRcaα decreased by a similar extent in plants inoculated with BSMV::Rca1 or BSMV::Rca2α, representing 25% of the abundance of this isoform compared with control plants (Figure 3a). The relative abundance of TaRcaβ was found to be decreased not only in plants treated with BSMV::Rca1 but, unexpectedly, also in plants treated with BSMV::Rca2α. Though, in the latter case, the decrease was less pronounced (50% of the abundance level in control plants) (Figure 3b). On the other hand, the different BSMV::Rca silencing constructs did not affect the abundance of Rubisco in the leaf samples, suggesting that the effect was specific to Rca (Figure 3c).

## 4 DISCUSSION

Silencing the individual *TaRca* genes and alternative transcripts of these genes using VIGS represents an early contribution towards understanding the regulation of *TaRca* gene expression. This understanding is important to inform strategies to alter the relative abundance of Rca isoforms with the aim to improve the regulation of Rubisco in dynamic light conditions and in response to global warming. In this study, the three *TaRca* transcripts have been effectively down‐regulated using VIGS.

The combined outcomes obtained here, using specific VIGS constructs to target the individual wheat Rca transcripts, suggest that co‐regulation of gene expression may occur at the level of transcription and translate to a decrease in non‐target TaRca protein isoforms. The qRT‐PCR results showed that the construct used for silencing the *TaRca1* gene also resulted in a down‐regulation of *TaRca2*, with decreased expression of both *TaRca2α* and *TaRca2β* compared with control plants (Figure 2). Similarly, the construct used for silencing the *TaRca2* gene also had a significant regulatory effect on the *TaRca1* gene, decreasing its expression relative to the control plants by a similar extent to that observed in BSMV::Rca1‐treated plants. Based on the si‐Fi prediction results (Figure S2), the VIGS fragments selected were unlikely to cause off‐target gene silencing. A minimum of 21 consecutive identical nucleotides (nt) between two genes would be needed to result in the production of siRNAs that target both *TaRca1* and *TaRca2* genes effectively (as dsRNA are cleaved into 21–24 nt long siRNAs; Baulcombe, 2004). The differences between the *TaRca1* and *TaRca2* gene sequences, with SNPs spaced more or less evenly along the gene sequence, would suggest that any individual VIGS construct used here would not be expected to silence both genes. This is particularly the case for the construct used for BSMV::Rca1. The construct used for BSMV::Rca2 was not predicted to be effective in selecting *TaRca1*, but with three blocks of 21 nt conserved between the two genes (Figure S1), the hypothesis cannot be ruled out that this construct could lead to off‐target silencing of *TaRca1*.

An alternative explanation for the down‐regulation of the untargeted *TaRca* transcript(s) using isoform‐specific sequences could potentially be transitive silencing. Transitive silencing of three endogenous catalase genes (*CAT1*, *CAT2*, and *CAT3*), accompanied by a knock‐down phenotype, was observed in Arabidopsis transgenic plants expressing an 800 nt region of the target catalase gene (*CAT2*), but only when the plants also contained an in trans silencing‐inducing transgene locus X_21_ (Bleys, Van Houdt, & Depicker, 2006). Transgenic plants harboring the 800 nt region *CAT2* locus alone did not exhibit transitive silencing of *CAT* genes (Bleys, Van Houdt, & Depicker, 2006; Bleys, Vermeersch, et al., 2006). Transitive silencing in rice (*Oryza sativa* L.) transformed with inverted‐repeat sequences (Miki et al., 2005) and in *N. benthamiana* Domin using a Potato virus X VIGS vector (Petersen & Albrechtsen, 2005) found that transitive silencing occurred when transgenes were targeted but not when endogenous gene sequences were the target. It has been hypothesized that transitive silencing only occurs when cleaved target sequences accumulate to a sufficiently high level—these then act as substrates for RNA‐dependent RNA polymerase (RDR6) to generate double‐stranded RNA upstream and downstream of the original target (Bleys, Van Houdt, & Depicker, 2006; Tang et al., 2003). Thus, while possible, it would seem unlikely that the accumulation of *TaRca1* and *TaRca2* cleavage products in our VIGS plants would be sufficiently high to trigger transitive silencing of the *Rca* gene family.

Treatment with BSMV::Rca2α showed a mild decrease in the expression of both *TaRca2α* and *TaRca2β* transcripts to a similar extent but did not show a significant decrease in *TaRca1β* (Figure 2). Again, the decrease in *TaRca2β* is unlikely to be due to off‐target silencing as the BSMV::Rca2α construct was designed to specifically target the C‐terminal extension of the *TaRca2α* transcript and in theory should not decrease the expression of *TaRca2β* or *TaRca1β*. Indeed, the expression of *TaRca1β* in these plants was comparable with that of control plants, but the expression of *TaRca2β*, which lacks the C‐terminal extension specific for *TaRca2α*, was lower than in control plants. The expression of *TaRca2α* was lower in plants treated with BSMV::Rca1 and BSMV::Rca2 than in plants treated with BSMV::Rca2α designed specifically to silence the *TaRca2α* transcript (Figure 2c). This suggests that the VIGS construct designed to target the end of the *TaRca2* mRNA, in the C‐terminal extension, was less effective in silencing the *TaRca2α* isoform than the BSMV::Rca2 construct designed in the middle of the *TaRca2* mRNA as suggested by the lower numbers of predicted effective siRNAs (Figure S2).

Rca silencing by VIGS has been carried out before in rice (Ding et al., 2006), using a VIGS construct targeted to silence the single, alternatively spliced, Rca gene in rice, and resulted in a decrease in the Rca mRNA levels to 6% of that in control plants. However, the effect of silencing on Rca protein abundance was not investigated. On the other hand, down‐regulation of a specific Rca isoform at the transcript and protein levels has been reported when RNAi was used in *Glycine max* L. (soybean) to decrease the expression of two genes that specifically encode the Rcaα isoform in this species (Harvey et al., 2022).

Decreased Rca at both transcript and protein levels was also seen in Arabidopsis plants treated with the plant hormone jasmonic acid (Shan et al., 2011). In general, in both bacteria and eukaryotes, the cellular concentrations of proteins correlate with the abundance of their corresponding mRNAs but not strongly. Discrepancies can be seen between transcript abundance and protein amount, and cross‐species studies indicate that only approximately 40% of the variation in protein concentration can be explained by the mRNA abundance (Vogel & Marcotte, 2012). Here, both gene expression (Figure 2) and protein abundance (Figure 3) decreased following treatments with the different silencing constructs but not to the same extent. This discrepancy can be appreciated by the lack of a significant correlation between gene expression and protein abundance for the TaRcaα and TaRcaβ isoforms in plants treated with specific VIGS constructs (Figure S4). This lack of correlation between mRNA accumulation and protein abundance for Rca isoforms in wheat suggests that the abundance of the Rca isoforms is post‐transcriptionally regulated (Perdomo et al., 2021).

In wheat flag leaves, TaRca1β represents only 1% to 2% of the Rca pool (Degen et al., 2021). Plants inoculated with the BSMV::Rca1 construct showed a decrease in abundance of the TaRcaβ (the sum of the TaRca1β and TaRca2β) by 76%, relative to the control. This large decrease in TaRcaβ implies a decrease in TaRca2β too, which is in line with the concomitant down‐regulation of the transcript levels observed between the two β isoforms encoded by the two different genes in wheat. Although the BSMV::Rca2α construct was designed to silence only the TaRcaα isoform encoded by *TaRca2* (Figures 1 and S1), a significant decrease in TaRcaβ abundance was also observed (Figure 3b). TaRca2β is the most abundant of the three Rca isoforms in wheat, representing approximately 84% of the Rca pool (Degen et al., 2021; Perdomo et al., 2021). Therefore, it is clear that silencing *TaRca2α* has also led to reduced levels of *TaRca2β*, an alternative transcript produced from the same gene, *TaRca2*. Treatment with BSMV::Rca2 decreased the protein abundance of both TaRcaα and TaRcaβ by 82% and 96%, respectively, relative to the control (Figure 3). The qRT‐PCR results showed relatively low expression levels of both *TaRca2* transcripts but a less significant decrease in the expression of *TaRca1β* (Figure 2). This supports the notion that most of the β isoform in wheat is produced from transcripts derived from the *TaRca2* gene.

Silencing the *TaRca* genes in this study had no impact on the abundance of Rubisco protein (Figure 3c). This is in contrast to previous studies in rice, in which overexpression of Rca was shown to decrease the amount of Rubisco and, consequently, the rate of photosynthesis (Fukayama et al., 2012; Suganami et al., 2020). These findings suggest that although excess Rca may repress Rubisco synthesis, reduced Rca does not trigger large changes in Rubisco abundance.

BSMV naturally infects barley and to a lesser extent wheat and several other monocots where it can cause severe stress to plants, and this is similar in the laboratory plants. Research into Rca protein levels in response to stress indicates post‐translational degradation; *Pinus halepensis* Mill. exposed to high levels of ozone or drought stress showed a marked decrease in Rca protein abundance (Pelloux et al., 2001). The BSMV::asGFP‐treated control and the silenced plants exhibited similar viral symptoms indicating that the effects seen here are not primarily due to a stress response. The silencing fragments used in this study could be used for further research using stable transgenic RNAi. The advantages of stable RNAi are that it offers constitutive silencing with no additional stress responses due to viral infection. Alternatively, novel mutagenic techniques could be used to specifically knock‐down the Rca genes. The CRISPR‐Cas system has been proving very promising; studies have shown specific and predictable mutagenesis of rice and wheat genes (Kumar et al., 2019; Shan et al., 2014).

In summary, the results obtained here indicate that silencing a specific transcript of wheat Rca by VIGS results in decreased expression of the other Rca transcripts, as well as the corresponding protein isoforms. In the wheat genome, *TaRca1* and *TaRca2* genes are located next to each other in tandem, which could explain the down‐regulation of the Rca isoforms encoded by the two genes. While the differential regulatory properties of Rca isoforms in wheat suggests scope for improving photosynthetic efficiency by altering the relative abundance of Rca isoforms (Perdomo et al., 2019; Scafaro, De Vleesschauwer, et al., 2019), the findings of the present study suggest that the manipulation of one isoform may impact on the expression of non‐target isoforms. Further research into the functional differences and significance of the diverse Rca isoforms is warranted and will inform strategies for improving the efficiency and climate resilience of photosynthesis.

## AUTHOR CONTRIBUTIONS

Elizabete Carmo‐Silva designed the experiments and supervised the project. Juan Alejandro Perdomo, Elizabete Carmo‐Silva, Wing‐Sham Lee, and Joanna C. Scales carried out the experiments. Wing‐Sham Lee and Kostya Kanyuka designed the VIGS silencing constructs. Juan Alejandro Perdomo analyzed the data. Juan Alejandro Perdomo and Elizabete Carmo‐Silva wrote the manuscript with contributions from all authors. All authors discussed the results, provided critical feedback, and contributed to the final manuscript.

## CONFLICT OF INTEREST STATEMENT

The authors declare that there are no competing interests associated with the manuscript.

## Supporting information


**Data S1.** Peer review

**Figure S1.** Wheat Rubisco activase (TaRca) sequence alignment highlighting the three regions targeted by the BSMV::Rca constructs
**Figure S2.** Off‐target predictions using siRNA‐Finder (Si‐Fi) software.
**Figure S3.** SDS‐PAGE gel and immunoblot detection and quantification of Rubisco and Rca respectively.
**Figure S4.** Relative gene expression and protein abundance correlations.
**Table S1.** Results of the statistical analysis to compare results obtained in the two experiments.
**Table S2.** MIQE guidelines for gene expression analyses.

## Data Availability

The dataset presented in this study is available in the Lancaster University's institutional repository system Pure.
