# Supplementary material for: Down‐regulation of wheat Rubisco activase isoforms expression by virus‐induced gene silencing
Source: Plant Direct. 2024 Apr 15;8(4):e583. doi: 10.1002/pld3.583 (PMC11018489; doi:10.1002/pld3.583)
Supplement: Supplementary file 2 — Figure S1. Wheat Rubisco activase (TaRca) sequence alignment highlighting the three regions targeted by the BSMV::Rca constructs Figure S2. Off‐target predictions using siRNA‐Finder (Si‐Fi) software. Figure S3. SDS‐PAGE gel and immunoblot detection and quantification of Rubisco and Rca respectively. Figure S4. Relative gene expression and protein abundance correlations. Table S1. Results of the statistical analysis to compare results obtained in the two experiments. Table S2. MIQE guidelines for gene expression analyses. [file PLD3-8-e583-s002.pdf]

Down-regulation of wheat Rubisco activase isoforms expression by virus-induced gene silencing

Juan Alejandro Perdomo[1,2*], Joanna C. Scales[3], Wing-Sham Lee[4], Kostya Kanyuka[4,5], Elizabete Carmo-Silva[1,3*]

[1] Lancaster Environment Centre, Lancaster University, Lancaster, LA1 4YQ, UK

[2] School of Pharmacy and Biomedical Sciences, University of Central Lancashire, Preston, PR1 2HE, UK

[3] Plant Biology and Crop Science, Rothamsted Research, Harpenden, AL5 2JQ, UK

[4] Biointeractions and Crop Protection, Rothamsted Research, Harpenden, AL5 2JQ, UK

[5] NIAB, 93 Lawrence Weaver Road, Cambridge, CB3 0LE, UK

*Contact E-mail: aperdomolopez@uclan.ac.uk and e.carmo-silva@lancaster.ac.uk

Figure S1. Wheat Rubisco activase (*TaRca*) sequence alignment highlighting the three regions targeted by the BSMV::Rca constructs

Figure S2. Off-target predictions using siRNA-Finder (Si-Fi) software.

Figure S3. SDS-PAGE gel and immunoblot detection and quantification of Rubisco and Rca respectively.

Figure S4. Relative gene expression and protein abundance correlations.

Table S1. Results of the statistical analysis to compare results obtained in the two experiments.

Table S2. MIQE guidelines for gene expression analyses.

```
                      10        20        30        40        50        60        70        80
              ....|....|....|....|....|....|....|....|....|....|....|....|....|....|....|....|
TaRca1β A     ATGGCTTCTGCTTTCTCGTCCACCGTTGGAGCTCCGGCGTCGACCCCGACCACCTTCCTCGGGAAGAAGGTGAAGAAGCA
TaRca1β B     ATGGCTTCTGCTTTCTCGTCCACCGTTGGAGCTCCGGCGTCAACCCCGACCACCTTCCTCGGGAAGAAGGTGAAGAAGCA
TaRca1β D     ATGGCTTCTGCTTTCTCATCCACCGTTGGAGCTCCGGCGTCGACCCCGACCACCTTCCTCGGGAAGAAGGTGAAGAAGCA
TaRca2α/β A   ATGGCTGCTGCCTTCTCCTCCACCGTCGGTGCCCCGGCTTCTACGCCGACCAACTTCCTCGGGAAGAAGCTCAAGAAGCA
TaRca2α/β B   ATGGCTGCTGCCTTCTCCTCCACCGTCGGTGCCCCGGCTTCTACGCCGACCAACTTCCTTGGGAAGAAGCTCAAGAAGCA
TaRca2α/β D   ATGGCTGCTGCCTTCTCCTCCACCGTCGGTGCCCCGGCTTCTACGCCGACCAACTTCCTCGGGAAGAAGCTCAAGAAGCA

                      90       100       110       120       130       140       150       160
              ....|....|....|....|....|....|....|....|....|....|....|....|....|....|....|....|
TaRca1β A     GGCCGGTGCGTTGAACTACTACCATGGTGGCAACAAGATCAACAATAGGGTGGTCAGGGCCATGGCGGCCAAAAAGGAAC
TaRca1β B     GGCCGGTGCGTTGAACTACTACCATGGTGGCAACAAGATCAAGAGTAAGGTGGTCAGGGCCATGGCGGCCAAAAAGGAAC
TaRca1β D     GGCCGGTACGTTGAACTACTACCATGGTGGCAACAAGATCAACAGTAGGGTGGTCAGGGCCATGGCGGCCAAAAAGGAAC
TaRca2α/β A   GGTGACCTCGGCCGTGAACTACCATGG---CAAGAGCTCCAAGGCCAACAGGTTCACAGTCATGGCAGCGGAAAACA---
TaRca2α/β B   GGTGACCTCGGCCGTGAACTACCATGG---CAAGAGCTCCAAGGCCAACAGGTTCACAGTCATGGCAGCGGAAAACC---
TaRca2α/β D   GGTGACCTCGGCCGTGAACTACCATGG---CAAGAGCTCCAAGGCCAACAGGTTCACGGTCATGGCAGCGGAAAACA---
                                                                                  BMSV::Rca1

                     170       180       190       200       210       220       230       240
              ....|....|....|....|....|....|....|....|....|....|....|....|....|....|....|....|
TaRca1β A     TTGACGAGGGCAAGCAGACCGATGCCGATCGGTGGAAGGGTCTCGCTTACGACATCTCCGATGACCAGCAGGACATCACG
TaRca1β B     TTGACGAGGGCAAGCAGACCAATGCCGATAGGTGGAAGGGTCTCGCTTACGACATCTCCGACGACCAGCAGGACATCACG
TaRca1β D     TTGACGAGGGCAAGCAGACCGATGCCGATCGGTGGAAGGGTCTCGCTTACGACATCTCCGACGACCAGCAGGACATCACC
TaRca2α/β A   TCGACGAGAAGAGG------AACACAGACAAGTGGAAGGGTCTTGCCTACGATATCTCCGACGACCAGCAGGACATCACC
TaRca2α/β B   TCGACGAGAAGAGG------AACACGGACAAGTGGAAGGGTCTTGCGTACGATATCTCCGACGACCAGCAGGACATCACC
TaRca2α/β D   TCGACGAGAAGAGG------AACACCGACAAGTGGAAGGGTCTTGCGTACGATATCTCCGACGACCAGCAGGACATCACC

                     250       260       270       280       290       300       310       320
              ....|....|....|....|....|....|....|....|....|....|....|....|....|....|....|....|
TaRca1β A     AGGGGGAAAGGCATCGTGGACTCCCTGTTCCAGGCCCCCATGGGCGACGGCACCCACGAGGCCATCCTGAGCTCCTACGA
TaRca1β B     AGTGGGAAAGGTATCGTAGACTCCCTGTTCCAGGCCCCCATGGGCGACGGCACCCACGAGGCCATCCTGAGCTCCTACGA
TaRca1β D     AGGGGGAAAGGTATCGTGGACTCCCTGTTCCAGGCCCCCATGGGCGACGGCACCCACGAGGCCATCCTGAGCTCCTACGA
TaRca2α/β A   AGAGGGAAGGGCATCGTGGACTCCCTCTTCCAGGCGCCCACGGGCGACGGCACCCACGAGGCCGTCCTCAGCTCCTACGA
TaRca2α/β B   AGAGGGAAGGGCATCGTGGACTCGCTCTTCCAGGCGCCCACGGGCGACGGCACCCACGAGGCCGTCCTCAGCTCCTACGA
TaRca2α/β D   AGAGGGAAGGGCATCGTGGACTCCCTCTTCCAGGCGCCCACTGGCGACGGCACCCACGAGGCCGTCCTCAGCTCATACGA

                     330       340       350       360       370       380       390       400
              ....|....|....|....|....|....|....|....|....|....|....|....|....|....|....|....|
TaRca1β A     GTACATCAGCCAGGGCCTGCGCAAGTACGACTTCGACAACACCATGGACGGGCTGTACATCGCCCCGGCGTTCATGGACA
TaRca1β B     GTACATCAGCCAGGGCCTGCGGAAGTACGACTTCGACAACACCATGGACGGCCTCTACATCGCCCCGGCATTCATGGACA
TaRca1β D     GTACATCAGCCAGGGCCTGCGGAAGTACGACTTCGACAACACCATGGACGGGCTCTACATCGCCCCGGCATTCATGGACA
TaRca2α/β A   GTACGTCAGCCAGGGACTCAAGAAGTACGACTTCGACAACACCATGGGAGGCTTCTACATCGCTCCTGCTTTCATGGACA
TaRca2α/β B   GTACGTCAGCCAGGGACTCAAGAAGTACGACTTCGACAACACCATGGGAGGCTTCTACATCGCTCCTGCTTTCATGGACA
TaRca2α/β D   GTACGTCAGCCAGGGACTCAAGAAGTACGACTTCGACAACACCATGGGAGGCTTCTACATCGCTCCTGCTTTCATGGACA

                     410       420       430       440       450       460       470       480
              ....|....|....|....|....|....|....|....|....|....|....|....|....|....|....|....|
TaRca1β A     AGCTCATCGTCCACCTCGCCAAGAACTTCATGACACTCCCCAACATCAAGGTCCCTCTCATCCTGGGTATCTGGGGAGGC
TaRca1β B     AGCTCATCGTCCACCTCGCCAAGAACTTCATGACACTCCCCAACATCAAGGTTCCTCTCATCCTGGGCATCTGGGGAGGC
TaRca1β D     AGCTCATCGTCCACCTCGCCAAGAACTTCATGACGCTCCCCAACATCAAGGTCCCTCTCATCCTGGGCATCTGGGGAGGC
TaRca2α/β A   AGCTTGTTGTCCATCTCTCCAAGAACTTCATGACCCTGCCCAACATCAAGATCCCACTCATCTTGGGTATCTGGGGAGGC
TaRca2α/β B   AGCTTGTTGTCCATCTCTCCAAGAACTTCATGACCCTGCCCAACATCAAGATCCCACTCATCTTGGGTATCTGGGGAGGC
TaRca2α/β D   AGCTTGTTGTCCATCTCTCCAAGAACTTCATGACCCTGCCCAACATCAAGATCCCACTCATCTTGGGTATCTGGGGAGGC

                     490       500       510       520       530       540       550       560
              ....|....|....|....|....|....|....|....|....|....|....|....|....|....|....|....|
TaRca1β A     AAGGGACAGGGCAAGTCGTTCCAGTGCGAGCTGGTGTTCGCCAAGATGGGCATCAACCCCATCATGATGAGCGCCGGAGA
TaRca1β B     AAGGGACAGGGCAAGTCGTTCCAGTGCGAGCTGGTGTTCGCCAAGATGGGCATCAACCCCATCATGATGAGCGCCGGTGA
TaRca1β D     AAGGGACAGGGCAAGTCGTTCCAGTGCGAGCTGGTGTTCGCCAAGATGGGCATCAACCCCATCATGATGAGCGCCGGAGA
TaRca2α/β A   AAGGGTCAAGGAAAATCCTTCCAGTGTGAGCTTGTCTTCGCCAAGATGGGCATCAACCCAATCATGATGAGTGCCGGAGA
TaRca2α/β B   AAGGGTCAAGGAAAATCATTCCAGTGCGAGCTTGTCTTCGCCAAGATGGGCATCAACCCAATCATGATGAGTGCCGGAGA
TaRca2α/β D   AAGGGTCAAGGAAAATCCTTCCAGTGTGAGCTTGTCTTCGCCAAGATGGGCATCAACCCAATCATGATGAGTGCCGGAGA

                     570       580       590       600       610       620       630       640
              ....|....|....|....|....|....|....|....|....|....|....|....|....|....|....|....|
TaRca1β A     GCTGGAGAGCGGCAACGCCGGCGAGCCGGCCAAGCTGATCCGGCAGAGGTACCGCGAGGCTGCCGACATTATCAAGAAGG
TaRca1β B     GCTGGAGAGCGGCAACGCCGGCGAGCCGGCGAAGCTGATCCGGCAGAGGTACCGCGAGGCTGCCGACATTATCAACAAGG
TaRca1β D     GCTGGAGAGCGGCAACGCCGGCGAGCCGGCCAAGCTGATCCGGCAGAGGTACCGCGAGGCGGCGGACATTATCAAGAAGG
TaRca2α/β A   GCTGGAGAGTGGCAACGCCGGAGAGCCAGCCAAGCTCATCAGGCAGCGGTACCGTGAGGCTGCAGACATGATCAAGAAGG
TaRca2α/β B   GCTGGAGAGTGGCAACGCCGGAGAGCCAGCCAAGCTCATCAGGCAGCGGTACCGTGAGGCTGCAGACATGATCAAGAAGG
TaRca2α/β D   GCTGGAGAGTGGCAACGCCGGAGAGCCAGCCAAGCTCATCAGGCAGCGGTACCGTGAGGCTGCAGACATGATCAAGAAGG
```

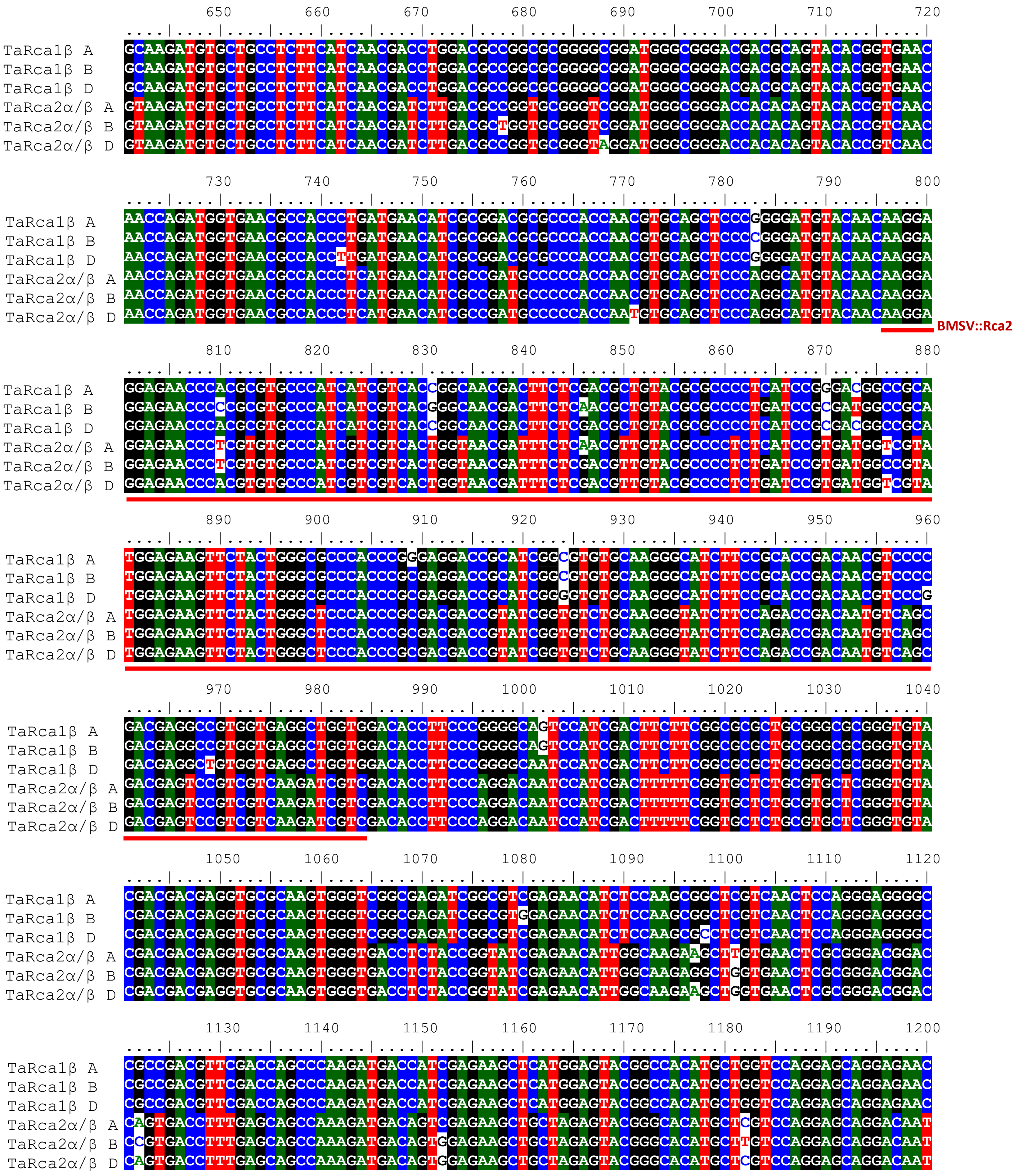
TaRca1β A
TaRca1β B
TaRca1β D
TaRca2α/β A
TaRca2α/β B
TaRca2α/β D
BMSV::Rca2

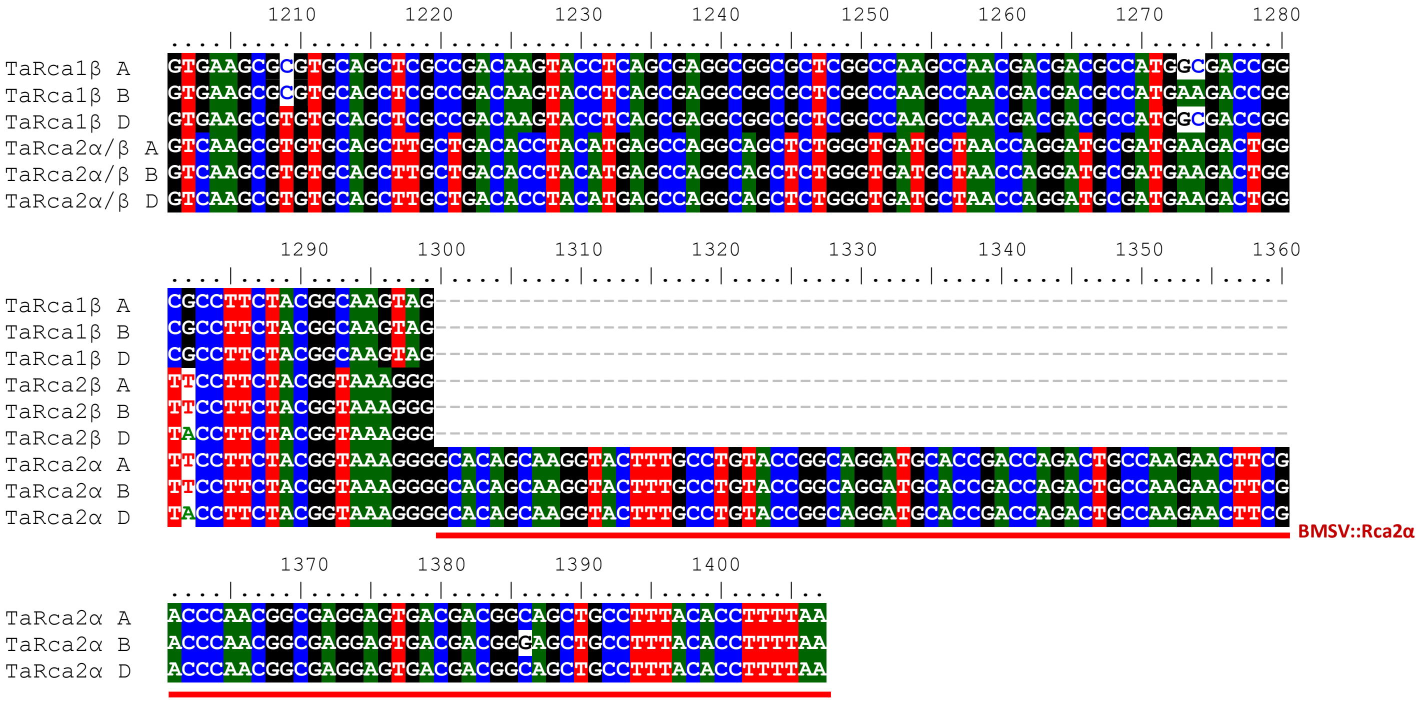

Figure 1. Wheat Rubisco activase (*TaRca*) sequence alignment highlighting the three regions targeted by the BSMV::Rca constructs

Nucleic acid sequence alignment of the three *TaRca* transcripts and splice variants from wheat (A, B and D genome; *TaRca1β*, *TaRca2β* and *TaRca2α*). Differences in nucleotide pairwise comparison between *TaRca1β* and *TaRca2α/TaRca2β* are highlighted through the use of different colours per nucleotide. The three regions targeted using VIGS are indicated by red coloured lines below the sequence alignment.

BSMV::Rca1

Targets	Total siRNA hits	Efficient siRNA hits
TaRca1 Genome A	97	42
TaRca1 Genome B	54	22
TaRca1 Genome D	24	9

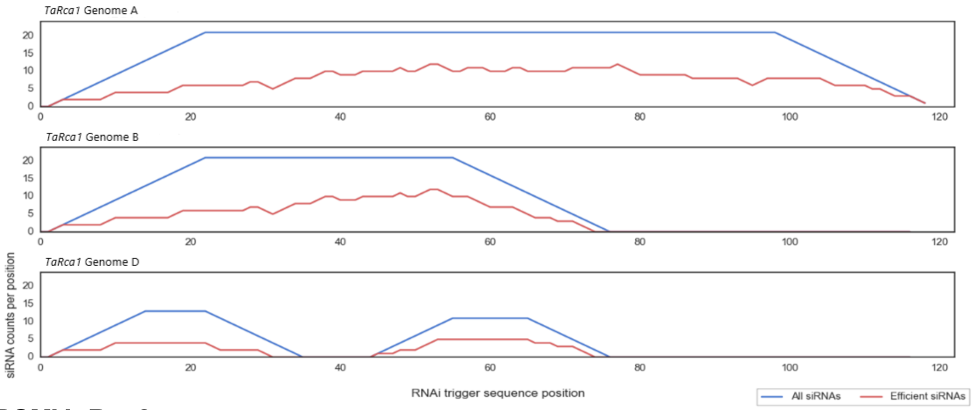

BSMV::Rca2

Targets	Total siRNA hits	Efficient siRNA hits
TaRca2 Genome A	169	92
TaRca2 Genome B	118	61
TaRca2 Genome D	115	65

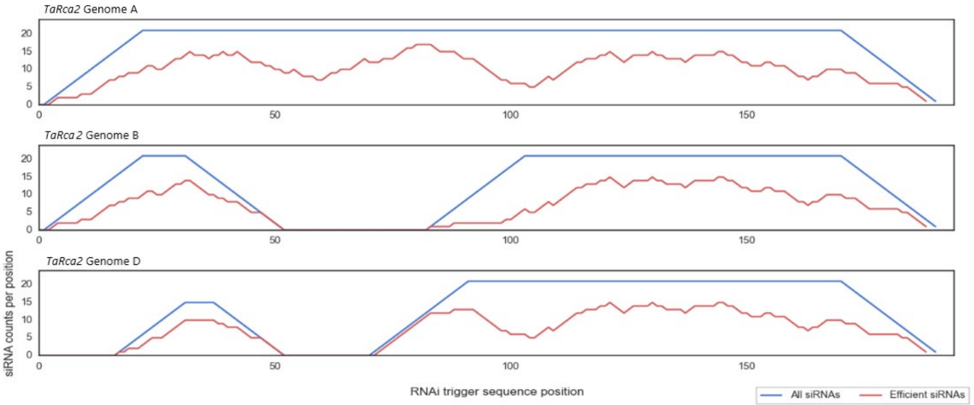

BSMV::Rca2α

Targets	Total siRNA hits	Efficient siRNA hits
TaRca2α Genome A	88	45
TaRca2α Genome B	67	37
TaRca2α Genome D	46	24

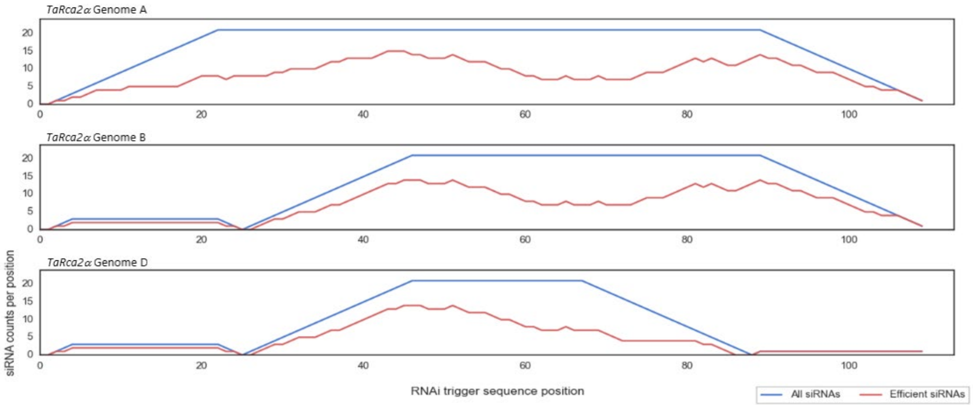

Figure S2. Off-target and silencing efficiency predictions using siRNA-Finder (si-Fi) software. The selection of Rca fragments used for generating the BSMV VIGS constructs was guided by si-Fi to select those predicted to be the most effective in silencing *Rca* target genes whilst having no effect on silencing off-target genes in wheat. The software outputs above show the si-Fi predictions for each of the selected Rca fragments. The blue line indicates the total number of siRNA hits; the red line corresponds to the number of siRNA hits that match the selected criteria for efficiency.

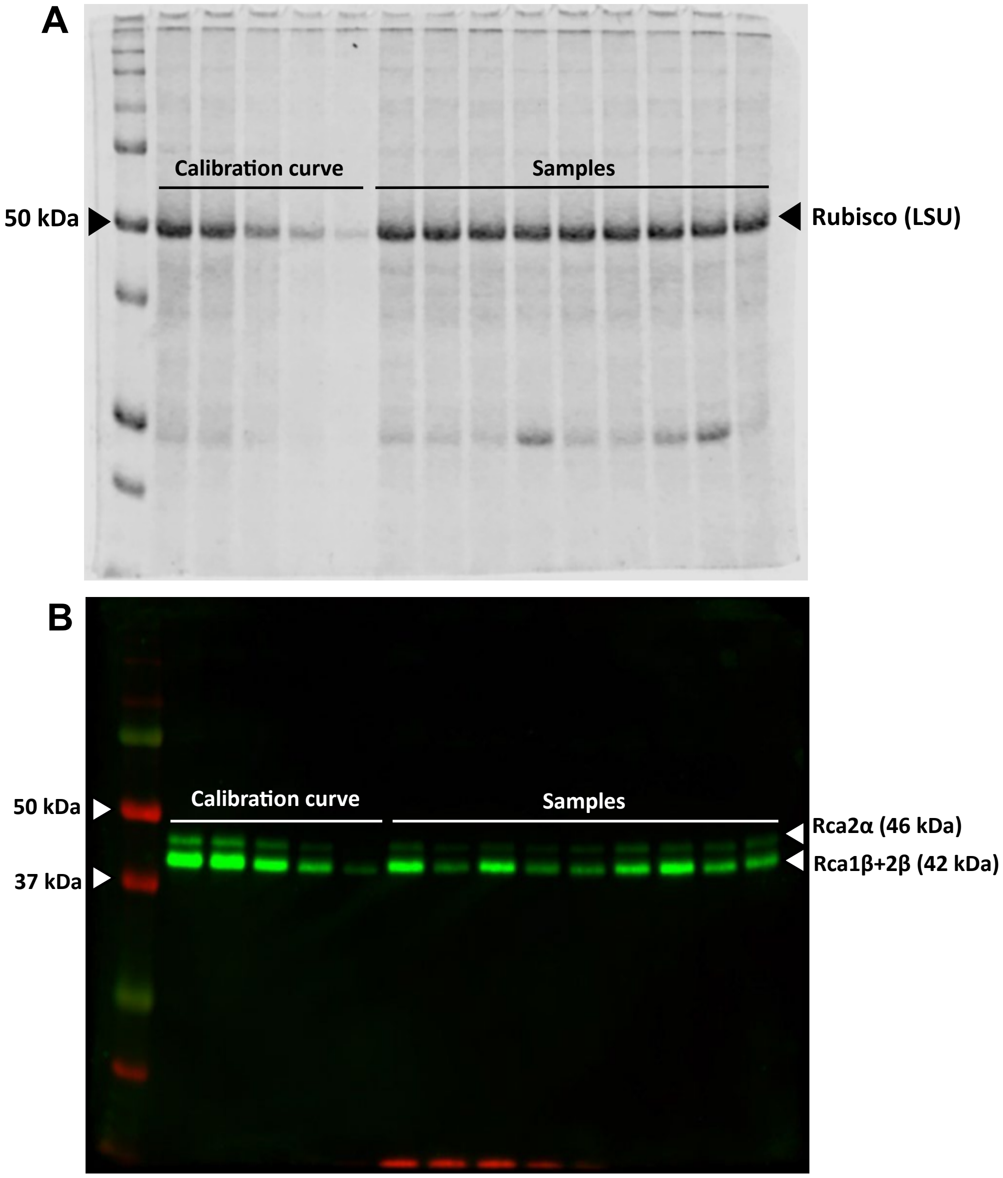

Fig. S3 SDS-PAGE gel and immunoblot detection and quantification of Rubisco and Rca respectively. A) Detection and quantification of Rubisco Large Subunit (LSU) by staining with Coomassie Blue. B) Detection and quantification of Rca α and β by immunoblotting using the polyclonal anti-Rca primary antibody. For Rca and Rubisco quantification a standard calibration curve was generated in each gel with a dilution series (1.2, 1.0, 0.5, 0.25, 0.1x) from the pool of BSMV::asGFP (a negative control) treated samples.

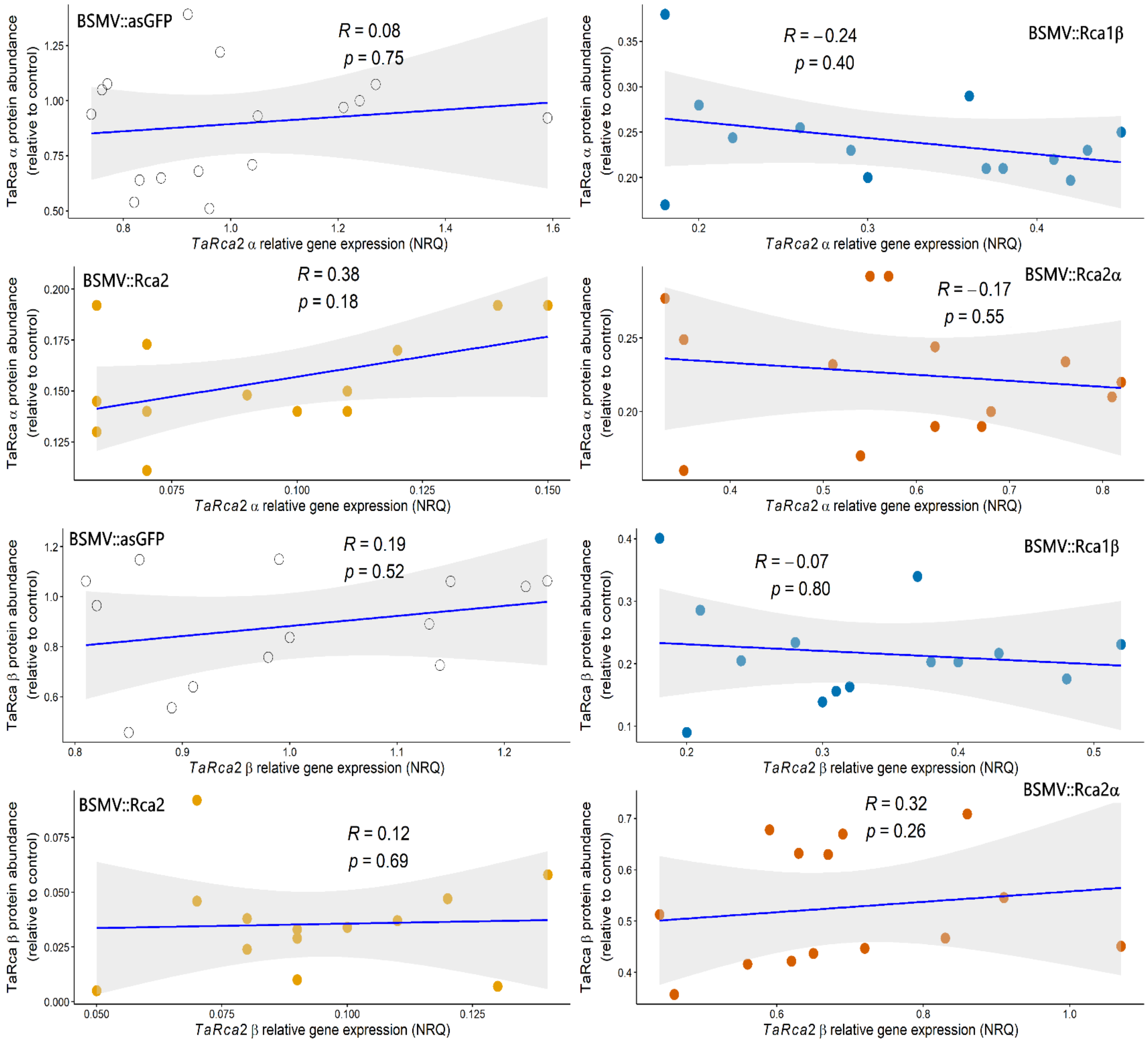

Figure S4. Relative gene expression and protein abundance correlations.
TaRcaα and *TaRcaβ* relative gene expression and the corresponding protein abundance correlations assessed in individual plants (*n* = 13–16) treated with the different BSMV VIGS constructs: BSMV::asGFP, BSMV::Rca1, BSMV::Rca2, and BSMV::Rca2α. There was no significant correlation between gene expression and protein amount of either of the two Rca isoforms following treatments with any of the different VIGS constructs (Spearman, P > 0.05).

Table S1. Results of the statistical analysis of the Rca genes expression and protein abundance data obtained in the two VIGS experiments.

Two independent experiments were performed to evaluate the effect of the VIGS silencing constructs on the gene expression and protein abundance of TaRca. Two-way ANOVA showed no significant effects of experiment ($P > 0.05$), and no significant interaction between experiment and construct ($P > 0.05$). Data for the two experiments was therefore analysed and presented together.

Construct	Rca Isoform or Rubisco	Measure	*P* value
BSMV::GFP	*TaRca1β*	Gene expression	1.000
BSMV::Rca1	*TaRca1β*	Gene expression	0.994
BSMV::Rca2	*TaRca1β*	Gene expression	0.999
BSMV::Rca2α	*TaRca1β*	Gene expression	0.999
BSMV::GFP	*TaRca2β*	Gene expression	1.000
BSMV::Rca1	*TaRca2β*	Gene expression	0.982
BSMV::Rca2	*TaRca2β*	Gene expression	1.000
BSMV::Rca2α	*TaRca2β*	Gene expression	0.999
BSMV::GFP	*TaRca2α*	Gene expression	1.000
BSMV::Rca1	*TaRca2α*	Gene expression	0.969
BSMV::Rca2	*TaRca2α*	Gene expression	1.000
BSMV::Rca2α	*TaRca2α*	Gene expression	0.862
BSMV::GFP	TaRcaα	Protein abundance	0.190
BSMV::Rca1	TaRcaα	Protein abundance	0.999
BSMV::Rca2	TaRcaα	Protein abundance	1.000
BSMV::Rca2α	TaRcaα	Protein abundance	0.984
BSMV::GFP	TaRcaβ	Protein abundance	0.541
BSMV::Rca1	TaRcaβ	Protein abundance	0.999
BSMV::Rca2	TaRcaβ	Protein abundance	0.999
BSMV::Rca2α	TaRcaβ	Protein abundance	0.199
BSMV::GFP	Rubisco Lsu	Protein abundance	0.710
BSMV::Rca1	Rubisco Lsu	Protein abundance	0.592
BSMV::Rca2	Rubisco Lsu	Protein abundance	0.645
BSMV::Rca2α	Rubisco Lsu	Protein abundance	0.339

Table S2. MIQE guidelines for gene expression analyses.

**MIQE guidelines** (as per Bustin *et al*. 2009)	
**Experimental design**	
Definition of experimental and control groups	Experimental group: wheat plants inoculated with three VIGS constructs BSMV::Rca1, BSMV::Rca2 and BSMV::Rca2α. Control group: wheat plants inoculated with the negative control BSMV::GFP construct.
Number within group	16 biological replicates per group (plant type), distributed in 2 experiments with 8 biological replicates from each experiment.
**Sample**	
Description	Leaf material from wheat plants.
Processing	Leaf segments were cut with scissors (which were cleaned with ethanol between samples) and then immediately snap frozen in liquid nitrogen and stored at -80°C until used.
**Nucleic acid extraction and quality assessment**	
Procedure	Frozen leaf material was ground with a chilled pestle and mortar to a fine powder. 15-20 mg were used for RNA extraction.
RNA extraction	Hot phenol method was used (Verwoerd *et al.,* 1989; Shinmachi *et al.,* 2010).
DNase treatment	RNA was dissolved in 150 µL water containing DNase for 15-30 min on ice and then incubated at 37°C for 30 min.
RNA concentration, purity	The total RNA concentration and quality was determined by measuring the absorbance at 230, 260 and 280 nm with a Nanodrop spectrometer (Thermo Fisher Scientific, Inc., UK) and following gel electrophoresis in a 1% (w/v) agarose gel. Pure RNA was defined as 260/280 around 2.0 and 260/230 above 1.8, respectively.
**cDNA synthesis**	
Reverse transcription reaction conditions	1 µg of RNA was added to a 12 µL reaction mixture containing 1 µL of oligo-dT primer and the mixture was incubated for 10 min at 70°C and then immediately cooled on ice. Reaction mixture contained dNTPs, RNase/DNase free water and SuperScript™ III RT/Platinum™ Taq as part of the Precision SuperScript III™ kit (ThermoFisher, UK).
**qPCR oligonucleotides**	
Primer sequences information	See Table 2 for details.
**qPCR protocol**	
qPCR conditions	50 °C for 2 min, 95 °C for 10 min, followed by 40 cycles of 95 °C for 15 s and 60 °C for 1 min
Melting curves analysis conditions	95 °C for 15 s, 60 °C for 1 min and 95 °C for 15 s
Reaction volume and cDNA amount	25 µL reaction volume. 40 ng cDNA per reaction.
Master mix	SYBRGreen (Platinum® SYBR® Green qPCR SuperMix-UDG w/ROX, Life Technologies, UK).
Primer concentration	250 nM
qPCR instrument	7500 Real-Time PCR machine (Applied Biosystems, Life Technologies, UK).
**qPCR validation**	
Specificity	Melt curve.

Primer efficiency	Primer efficiency was estimated using the linear phase of all individual reaction amplification curves (Ramakers *et al.,* 2003) and calculated using the LinReg-PCR package (Tuomi *et al.,* 2010).<br><br>*TaRca1β* 1.91<br>*Rca2β* 1.92<br>*Rca2α* 1.89<br>*TaTIP41* (reference gene 1) 1.87<br>*TaCDC48* (reference gene 2) 1.93
**Data analysis**	
qPCR analysis programme	ExpressionSuite™ Software (ThermoFisher, UK).
Cq determination	Followed protocol in the Manual.
Normalisation method	As described by Rieu and Powers (2009).
Statistical method	Kruskal–Wallis test was used to test the statistical significance in gene expression among the different constructs. A post hoc test using the Fisher's least significant difference criterium was used for multiple pairwise comparisons.

References

Bustin *et al*. (2009) The MIQUE guidelines: Minimum information for publication of quantitative real -time PCR experiments. Chemical Chemistry 55:611–622.

Ramakers C, Ruijter, Lekanne Deprez RH, Moorman AFM (2003) Assumption-free analysis of quantitative real-time polymerase chain reaction (PCR) data. Neurosci Lett 339:62–66

Rieu I, Powers SJ (2009) Real-time quantitative RT-PCR: design, calculations, and statistics. Plant Cell 21:1031–1033.

Shinmachi F, Buchner P, Stroud JL, Parmar S, Zhao FJ, Mcgrath SP, Hawkesford MJ (2010) Influence of sulfur deficiency on the expression of specific sulfate transporters and the distribution of sulfur, selenium, and molybdenum in wheat. Plant Physiology 153:327–336.

Tuomi JM, Voorbraak F, Jones DL, Ruijter JM (2010) Bias in the Cq value observed with hydrolysis probe based quantitative PCR can be corrected with the estimated PCR efficiency value. Methods 50:313–322

Verwoerd TC, Dekker BMM, Hoekema A (1989) A small-scale procedure for the rapid isolation of plant RNAs. Nucleic Acids Res 17:2362.
