# Supplementary material for: Down‐regulation of wheat Rubisco activase isoforms expression by virus‐induced gene silencing
Source: Plant Direct. 2024 Apr 15;8(4):e583. doi: 10.1002/pld3.583 (PMC11018489; doi:10.1002/pld3.583)
Supplement: Supplementary file 1 — Data S1. Peer review [file PLD3-8-e583-s001.pdf]

Plant Direct
Open Access

Down-regulation of wheat Rubisco activase isoforms expression by virus2 induced gene silencing Juan Alejandro Perdomo1,2*, Joanna C. Scales3 , Wing-Sham Lee4 3 , Kostya Kanyuka4,5, Elizabete Carmo-Silva1,3*

Decision Letter Round 1:

August 28, 2023

Dr. Alejandro Perdomo Lopez
University of Central Lancashire
School of Pharmacy and Biomedical Sciences
Maudland Building office 149
Preston PR1 7DP
United Kingdom

RE: Down-regulation of wheat Rubisco activase isoforms expression by virus-induced gene silencing

Dear Dr. Perdomo Lopez:

Thank you for submitting to Plant Direct. All required reviews have been returned and we have now finished our evaluation of your manuscript. In light of the reviewers' and editor's comments, further revisions are needed before the paper can be accepted for publication in Plant Direct.

Please view the editors' and reviewers' comments below and use their suggestions as a guide while you work on your revision.

When uploading the revised version of this article, please be sure to include the following:

-A word document that contains your response to the reviewers. You should respond to each reviewer comment and note the changes made to the manuscript. If you do not agree with a reviewer's comment and choose not to make a suggested revision, please explain why. Please try to provide as complete an answer as possible to each reviewer's criticisms in the "response to reviewers" section.
-A tracked changes document
- A clean version of the latest version of the manuscript

One reviewer raised important questions about methodology that should be addressed/ justified. Additionally, authors should make sure that all assumptions are supported by the data.

To upload your revision, please click the link below.
https://plantdirect.msubmit.net/cgi-bin/main.plex?el=A2Lr6bh1A6oBl6I4A9ftd1rD5gnz8aYXlA5c5vVbvwZ

In order to provide as timely a service as possible, we ask that your revision is resubmitted within three months after receipt of this request. If an extension is needed, please send a request, along with a brief explanation, to the editorial office at plantdirect@wiley.com .

Thank you very much for giving us an opportunity to review your work. I look forward to receiving the next version.

Sincerely,
Ana Fortes

-- Editor comments:
One reviewer raised important questions about methodology that should be addressed/ justified. Additionally, authors should make sure that all assumptions are supported by the data.

-- Reviewer comments:
Reviewer #1:

In this paper, the authors try to silence independently the three Rubisco activase isoforms in the hexaploid wheat species. VIGS was chosen to lead this approach, as this is an effective way of inducing silencing, although accompanied by some viral symptoms. The authors observe that their use of isoform-specific sequences on either the Rca1 or Rca2 genes to induce silencing leads to the reduction of the targeted transcript and corresponding protein accumulation, however the reduction is also observed on the untargeted transcript. They conclude that the two Rca genes are coregulated. The authors also examine the effects of reduced expression of Rca on Rubisco accumulation, but do not observe any effect.
In my opinion, in its current state, the conclusions are not supported strongly enough by the experiments.
The main conclusion states that Rca paralogs are coregulated because silencing one Rca isofom affects the expression of the second. This conclusion seems an overstatement as the results may be explained

by other hypotheses related to the mechanism of VIGS silencing. Although VIGS silencing is widespread, it is usually not used to silence a specific member of a gene family. This is because controlling its effect on a gene family is difficult. First, the region used to convey the silencing may act in trans on the other paralogs because it displays too much homology to the other paralogs. This point is raised by the authors in the discussion, but according to them, this is not the case for the regions used in this work. Indeed the regions used to silence specifically Rca1 would seem divergent enough not to target Rca2. Similarly in the case of the Rca2 alpha-specific form, the region used is not present in the Rca1 gene. However, the region used to silence both isoforms (Rca2 alpha and beta) stemming from the Rca2 gene, shows several conserved blocks of more than 23 nt (at least 3) in both Rca1 and Rca2 paralogs. Silencing in trans could thus occur leading to downregulation of all Rca isoforms as observed here. Indeed, this construct is the most efficient one, resulting in transcript and protein reduction for all three isoforms. The second point that could be raised here is that VIGS can induce "transitive silencing" (Bleys A, Van Houdt H, Depicker A. Down-regulation of endogenes mediated by a transitive silencing signal. RNA. 2006 Sep;12(9):1633-9. doi: 10.1261/rna.108106, for a review: Sanan-Mishra et al., Front. Plant Sci., https://doi.org/10.3389/fpls.2021.610283)). Transitive silencing could be completely compatible with the observations made here: silencing of Rca1 could induce secondary sRNA downstream of the targeted sequence, whose sequence would be homologous enough to induce the silencing of the Rca2 paralog. Curiously, this possibility is not discussed at all in the manuscript. To support the coregulation hypothesis raised by the authors, it would be necessary to be able to rule out whether the effect could not be due to transitive silencing, which could be experimentally assessed through the detection of these sRNA.
Beside this major concern about the methodology used, additional points would need further examination:
- line 40: correct "Rubisco binding protein" to "Ribulose 1,5 bisphosphate"
-result section: Table 2: the primers used for qRT-PCR for Rca2b seem to be incorrectly described. The reverse primer is the one used for Rca2alpha, the forward primer could not be found within the provided sequence. Please clarify this point. It would be easier for the reader to have a visual localization of the primers used on the sequence provided in figure S1.
- Figure S1: the 3' end sequence of Rca1betaD (bp 1281 to 1300) is curiously close to Rca2 beta A and B, while the Rca2beta D is close to the Rca1beta A and B. Please check whether this is correct or a problem in the alignment. Also, the VIGS targeted regions are indicated in the legend to be below the sequence. However this is not the case for rca1beta and rca2, where the red line is incorrectly placed.
- line 229: authors indicate that Rca1 beta level remains unchanged upon silencing with the Rca2alpha specific region, shown to induce Rca 2alpha and beta reduction (median around 60% of levels observed in the control. This is based on the statistical analysis of the Rca1beta transcript between the control and VIGS-Rca2alpha silenced plants, but may seem a bit too strong according to the figure, in which the Rca1beta median is around 80% compared to the control.
- Discussion section; Line 272: please add BSMV::Rca2alpha showed a MILD decrease...
- line 313: overstatement: in line with the reduction of transcript levels (instead of coregulation)
- Rubisco abundance is measured by Ponceau stain. To be able to discuss Rubisco accumulation, this

method seems very imprecise, and standard immunoblotting with commercially available Rubisco antibodies would seem more appropriate.
- lines 349-351: as mentioned in the general points, this conclusion is poorly supported by the data provided.

Minor points:
- line 84: the authors claim that little is known concerning the regulation of Rubisco activase isoforms although they showed in a previous report that Rca1 is induced by heat. This could be mentioned here.
- line 128: reference to Yuan et al., 2011 is not found in the bibliography section.
- MM section, line 177 indicated that ten biological replicates have been collected while table S2 indicated 8.

Reviewer #2:

• Please explain critically the potential application of the current findings at large scale in agricultural fields. This should be added in the conclusion section.
• Please cite recent references (last 5-10 years) in the Introduction section.
• L40: Please check RuBP full form.
• L40-43: It would be better to break the complex sentence into two like "However,....conformation." "One of its inhibitors is its substrate, RuBP ... Rubisco".
• Lack of use of articles in several places, for example, in L41 "the" unproductive binding, "the" active sites; in L107 "the" production. Please check the whole manuscript and do the necessary corrections.
• L80-81: Use of "the more highly expressed" is not technically sound.
• L84-85: There are some recent papers available on regulation of the Rubisco activase isoforms expression in wheat, especially under heat stress.
• Line 95: Please use either "2-3 weeks" or "two to three weeks" instead of "two-three weeks".

Decision Letter Round 2:

March 21, 2024

Dr. Alejandro Perdomo Lopez
University of Central Lancashire
School of Pharmacy and Biomedical Sciences
Maudland Building office 149
Preston PR1 7DP

United Kingdom

MSID: 2023-01232R2
MS TITLE: Down-regulation of wheat Rubisco activase isoforms expression by virus-induced gene silencing

Dear Dr. Alejandro Perdomo Lopez:

I am pleased to inform you that your manuscript "Down-regulation of wheat Rubisco activase isoforms expression by virus-induced gene silencing" has been accepted for publication in Plant Direct.

Your article will appear online in the next available issue of Plant Direct. To ensure your article gets published as quickly as possible, please pay attention to the steps detailed below. We have found that most of the delays happen at this stage, especially at the payment stage, so please respond as quickly as possible when prompted.

License Agreement: Once your article has been accepted it will move to Production and undergo admin and file checking - you may receive an email with any queries we have at this stage. When all required items are received by the publisher and queries resolved, the corresponding author will receive an email from Wiley's Author Services system which will ask them to log on at https://authorservices.wiley.com/bauthor and will present them with the appropriate license for completion. Your article cannot be published until both the signed license agreement and payment of the article fee have been received.

Payment of the Open Access Article Publication Fee: All articles published in Plant Direct are fully open access: immediately and freely available to read, download and share. Plant Direct charges a publication fee to cover the publication costs. The corresponding author for this manuscript should have already received a quote from the payments team (cs-openaccess@wiley.com) with the estimated article publication fee; please email cs-openaccess@wiley.com if this has not been received. The corresponding author should log on to the Wiley Author Services site, where the publication fee can be paid by credit card or an invoice. Pro Forma can also be requested. Payment of the publication charge must be received before the article will be published online.

Proofs: You will have the opportunity to look over your paper once more when you receive the author proofs for your article. The proofs will be with you in approximately two weeks. Please note that, in addition to publishing reviewer comments, the author's responses to review comments will also be published alongside the final version of the paper. If you would not like the author's responses to be published, please contact the editorial office at plantdirect@wiley.com .

Promotion of your article: You can help your research get the attention it deserves! Check out Wiley's free Promotion Guide for best-practice recommendations for promoting your work at www.wileyauthors.com/eeo/guide. And learn more about Wiley Editing Services which offers professional video, design, and writing services to create shareable video abstracts, infographics, conference posters, lay summaries, and research news stories for your research at www.wileyauthors.com/eeo/promotion.

Thank you again for your contribution to Plant Direct. If you have any questions, feel free to contact the editorial office at plantdirect@wiley.com .

Sincerely,

Ana Fortes

Ana Fortes

Editor, Plant Direct

Author's response:

Down-regulation of wheat Rubisco activase isoforms expression by virus-induced gene silencing Perdomo et al. Plant Direct – Response to Reviewers Editor: The reviewer and myself only recommend minor changes before publication. AUTHORS: We are thankful for the candid and constructive feedback on our manuscript. Below we provide answers and explain how we have addressed each of the reviewer's comments. Reviewer #1: In this revised manuscript, the initial goal to manipulate the relative expression of Rubisco activase isoforms is more clearly addressed. The text introduces additional recent references, helping the reader to better understand the question. Concerning the methodology, the alternative hypotheses suggested by my previous review are introduced, although presented as "unlikely". In absence of further experimental evidence, these are in my opinion not to be disregarded, but I agree that the overall message is now tuned down. The suggested minor corrections have been implemented. Still, there are sentences that need to be clarified: AUTHORS: We appreciate the candid and constructive feedback. We addressed the suggested minor changes. - line 88, the sentence "differences in the TaRca transcript sequences were sufficient to enable the use of VIGS to knock-down expression of each of the individual transcripts ..." is not correct. This was the goal, but whether the differences were sufficient has not been shown. Please replace with the more general term "used". Concerning the part about "each of the individual transcript", turningdown the Rca2beta isoform specifically has not been attempted ... AUTHORS: Thank you, changed accordingly. - In the result section,

line 216, please replace the sentence "three different constructs were developed to specifically reduce the abundance of the Rubisco activase isoforms independently using VIGS" by something like "three different constructs were developed to specifically reduce the relative abundance of the different Rubisco activase isoforms independently using VIGS. AUTHORS: Thank you, changed accordingly. - same idea in the abstract, line 22: remove "independently". AUTHORS: Thank you, changed accordingly. - in the discussion part, line 283 (278): the presence of blocks of conserved 21 nt sequence between the TaRca1 and TaRca2 genes is now mentioned for the region used to silence Rca2, but not for the region used to silence Rca1, whereas the nt from positions 213 to 239 are conserved. So offtargeting of Rca2 by VIGS-Rca1 could occur and should be mentioned as such, and not be restricted to the off-targeting of Rca1 by Vigs-Rca2. Please revise the paragraph accordingly. AUTHORS: We believe the reviewer misinterpreted our supplementary figure; the region used to silence Rca1 ends at position 185, it does not include the conserved sequence they mention between nt 213-239. We have considered editing the text in the manuscript and changing the supplementary figure, however, we decided against as the text acknowledges this is something to consider, and the figure caption states that the red coloured lines are placed below the sequences. About the results: the VIGS construct targeting the Rac2alpha isoform appears as the least effective one in decreasing the Rca2alpha transcript and this is discussed in the discussion (lines 316-320). However in this case there is a much stronger effect at the level of Rca2alpha protein accumulation. While an extensive discussion is presented to explain possible discrepancies between mRNA levels and protein levels (line 332-341), this could also be due to a limitation by the antibody or quantification method used. Regretfully, raw data about protein accumulation of the samples versus a standard is not presented at all. We have to trust the authors that the antibody response is linear, and the gels are well loaded. Presenting some of the blots in supplemental data would allow to rule this hypothesis. AUTHORS: Thank you for the suggestion. A new figure has been added to the supplementary data (Fig. S3) with one of the gels and one of the blots used to quantify Rubisco and Rca in the experiment. - lines 325-327 (320-324): the fact that silencing of a specific isoform with RNAi is specific of the alpha isoform in soybean should be explained further: is there a similar gene organization with the alpha form stemming from alternative splicing as in wheat? AUTHORS: The sentence was edited to clarify that in Harvey et al. 2022 the two genes that only encode the Rcaα isoform were silenced using RNAi. - lines 356-359: the two sentences are redundant, remove second sentence "However, in this case..." AUTHORS: Thank you, changed accordingly.
